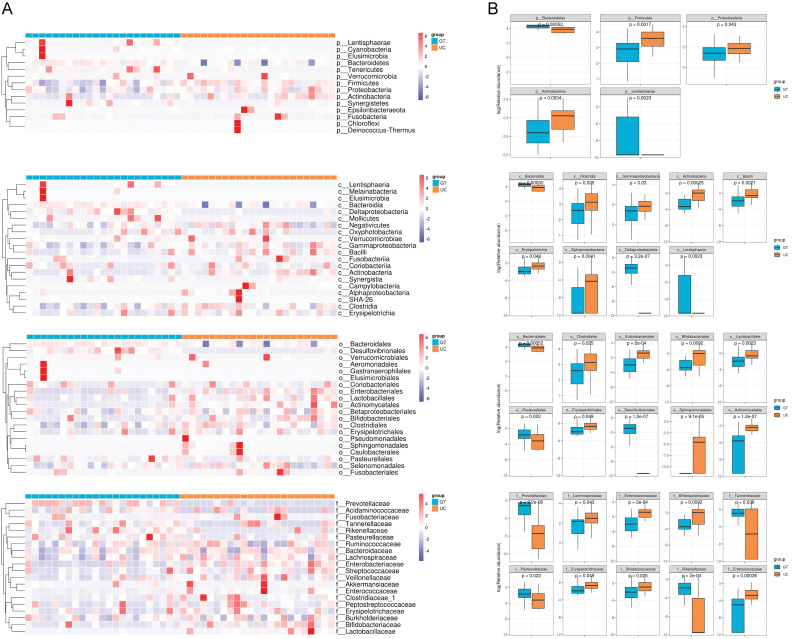# Gut microbiota-based discriminative model for patients with ulcerative colitis: A meta-analysis and real-world study

**DOI:** 10.1097/MD.0000000000037091

**Published:** 2024-03-08

**Authors:** Rong Zhang, Jing Chen, Li Liu, Xiankun Li, Changwei Qiu

**Affiliations:** a Department of General Surgery, The Third People’s Hospital of Chengdu, Chengdu 610014, Sichuan Province, China; b Department of Gastroenterology, The People’s Hospital of Dujiangyan, Dujiangyan 611830, Sichuan Province, China; c Department of Gastroenterology, The Third People’s Hospital of Chengdu, Chengdu 610014, Sichuan Province, China; d Department of Pharmacy, The People’s Hospital of Dujiangyan, Dujiangyan 611830, Sichuan Province, China.

**Keywords:** 16S rRNA gene sequencing, diagnostic efficacy, gut microbiota, meta-analysis, ulcerative colitis

## Abstract

Gut microbiota directly interacts with intestinal epithelium and is a significant factor in the pathogenesis of ulcerative colitis (UC). A meta-analysis was performed to investigate gut microbiota composition of patients with UC in the United States. We also collected fecal samples from Chinese patients with UC and healthy individuals. Gut microbiota was tested using 16S ribosomal RNA gene sequencing. Meta-analysis and 16S ribosomal RNA sequencing revealed significant differences in gut bacterial composition between UC patients and healthy subjects. The Chinese UC group had the highest scores for *Firmicutes, Clostridia, Clostridiales, Streptococcaceae,* and *Blautia*, while healthy cohort had the highest scores for *P-Bacteroidetes, Bacteroidia, Bacteroidales, Prevotellaceae*, and *Prevotella_9*. A gut microbiota-based discriminative model trained on an American cohort achieved a discrimination efficiency of 0.928 when applied to identify the Chinese UC cohort, resulting in a discrimination efficiency of 0.759. Additionally, a differentiation model was created based on gut microbiota of a Chinese cohort, resulting in an area under the receiver operating characteristic curve of 0.998. Next, we applied the model established for the Chinese UC cohort to analyze the American cohort. Our findings suggest that the diagnostic efficiency ranged from 0.8794 to 0.9497. Furthermore, a combined analysis using data from both the Chinese and US cohorts resulted in a model with a diagnostic efficacy of 0.896. In summary, we found significant differences in gut bacteria between UC individuals and healthy subjects. Notably, the model from the Chinese cohort performed better at diagnosing UC patients compared to healthy subjects. These results highlight the promise of personalized and region-specific approaches using gut microbiota data for UC diagnosis.

## 1. Introduction

Ulcerative colitis (UC) is a disorder of unknown cause characterized by inflammation starting in the rectum and spreading to the rest of the colon mucosa.^[1,2]^ UC therapy can be divided into 2 categories: induction and maintenance therapies. The primary objective of treatment is to attain or maintain clinical and endoscopic remission, reduce the incidence of complications, and improve patient quality of life.^[1,3]^ UC is classified into 3 categories based on severity: mild, moderate, or severe. Lesions associated with UC can be categorized as proctitis, left-sided colitis, or pancolitis. Treatment strategies for UC vary depending on the severity of the disease and the range of lesions.^[3]^ Nonbiological drugs are critical components of UC treatment, and biological drugs have become increasingly effective in recent years.^[4,5]^ Surgery remains the sole treatment for UC, with approximately 15% of patients necessitating coloproctectomy. Clinically, surgery is recommended only in specific cases, including refractory UC or neoplastic lesions.^[3,5]^

Although several studies have investigated the pathophysiology of UC, the exact cause remains largely unknown. Clinical symptoms of UC are typically marked by recurring bloody stools and abdominal pain accompanied by the presence of mucus.^[6,7]^ The initial events in the development of UC involve damage to the mucosal barrier, changes in gut microbiota, and an abnormal immune response in the intestine. UC pathogenesis is commonly attributed to the interaction between environmental and host factors, which increases the likelihood of developing the disease. Nongenetic factors, including epigenetics, abnormal adaptive immune responses, and epithelial barrier dysfunction, may also play a role in the development of UC.^[8–10]^

Current research indicates that the development of UC is influenced by various factors, including genetics, environment, immunity, and the microbiome. High-throughput sequencing has provided evidence that intestinal dysbiosis, an imbalance in gut bacteria, plays a significant role in the onset and progression of UC.^[11,12]^ Studies have indicated that individuals with UC experience a decrease in the diversity and stability of their gut microbiota. The reduction of protective bacteria, such as *Ruminococcaceae* and *Chaetobacteriaceae*, leads to a decrease in the production of short-chain fatty acids, which have anti-inflammatory effects on intestinal epithelial cells, macrophages, and dendritic cells.^[13]^ Conversely, there is a significant increase in the abundance of proinflammatory microorganisms such as *Enterobacteriaceae* and *Fusobacteriaceae*.^[14,15]^ Intervention studies have shown that fecal microbial transplantation from healthy donors can alleviate symptoms in patients.^[16–18]^ This treatment method has been found to contribute to UC remission by restoring intestinal microbial diversity, including short-chain fatty acid-producing bacterial species from donor feces.^[16,18]^ One of the primary effects of intestinal microbiome disorders in UC is dysfunction of the intestinal epithelial barrier or epithelial dysfunction, which can trigger susceptibility to UC. Further investigation into the distribution of intestinal flora in patients with UC and its correlation with patient prognosis can aid in identifying the risk factors and prognostic targets associated with UC.

## 2. Methods and materials

We conducted a systematic search of previous publications in the National Center for Biotechnology Information, Excerpta Medica Database, and Web of Science databases until May 2020, following the Preferred Reporting Items for Systematic Reviews and Meta-Analyses guidelines. Our search strategy included the terms “ulcerative colitis,” “gut microbiota,” “human,” and “16S ribosomal RNA (rRNA).” Additionally, we manually checked all retrieved articles and identified 4 relevant articles from the United States. The inclusion criteria were as follows: (1) fecal samples were collected from patients diagnosed with UC and healthy individuals. (2) Next-Generation Sequencing was used to sequence the 16S rRNA gene. (3) Raw sequencing data, barcodes, and metadata were all available for analysis.

### 2.1. Data processing of the included datasets

The included studies utilized 16S rRNA gene sequencing on either the Illumina (MiSeq or HiSeq) or NextSeq 500 platforms. Despite the different sequencing procedures for the 16S rRNA gene in each platform, a uniform analysis process was employed to mitigate the potential impact of these differences. Data quality filtering was performed using USEARCH (http://www.drive5.com/usearch/). Paired-end reads were assembled using Fast Length Adjustment of SHort reads (v1.2.11). The assembled sequences and single-end reads were removed using Cutdapt (v1.13) and quality filtered with a minimum quality score of 20. The SILVA (from Latin silva, forest, http://www.arb-silva.de) database (v132) was used to cluster the data into operational taxonomic units (OTUs) with 97% identity using closed-reference clustering. To ensure accurate classification and diversity analyses, we excluded samples with sequencing depths of < 10,000 reads in the OTU table. Additionally, we rarefied the OTU table to the lowest number of reads per sample for each analysis.

### 2.2. Inclusion and exclusion criteria for healthy cohort and patients with UC in China

The study enrolled 46 participants, 23 of whom were diagnosed with UC and 23 who were not. Individuals who did not provide consent; those with a history of oncology; and those who had used antibiotics, probiotics, or prebiotic supplements within 3 months prior to data collection were excluded. The diagnosis of UC was based on clinical, endoscopic, and histological criteria. The participants’ characteristics are listed in Table 1. The study was reviewed and approved by the Ethics Committee of the Third People’s Hospital of Chengdu. All participants provided written informed consent.

**Table 1:** The clinicopathological factors of HC (*n* = 23) patients and UC (*n* = 23).

Characteristics	HC of cases (%)	UC
Age (yr)	42.36 ± 12.48	53.86 ± 9.25
Gender		
Male	12	11
Female	11	12
BMI (kg/m^2^)	24.13 ± 2.74	23.58 ± 2.28
Smokers		
Yes	10	14
No	13	9
Alcohol consumers		
Yes	7	12
No	15	11
Duration (yr)		5 ± 4.25

BMI = body mass index, HC = healthy control, UC = ulcerative colitis.

### 2.3. DNA extraction from feces and 16S rRNA gene sequencing in China dataset

After educating each group on their respective dietary restrictions, normal dietary habits were maintained for over a week. Following this, 1 g of feces was collected and promptly stored in 5 mL tubes with a preservation solution at 4 °C.

DNA was extracted from each sample using the QIAamp Rapid DNA Stool Mini Kit (QIAGEN, Hilden, Germany) following the manufacturer’s instructions. A total of 0.25 g sample was used for the extraction process. DNA concentration was measured using a spectrophotometer. DNA integrity and length of the DNA was assessed using 1% agarose gel electrophoresis. The V4 region of the 16S rRNA gene was amplified using universal primers, where the 16S forward primer sequence was 5′-GTGCCAGCMGCCGCGGTAA-3′ and the reverse primer sequence was 5′-GGACTACNVGGGTWTCTAAT-3′. Amplicons were sequenced using the Illumina HiSeq platform. High-quality clean reads were generated by merging and quality control using Fast Length Adjustment of SHort reads software, followed by filtering chimeric sequences (Chimera_check). OTU clustering was performed using USEARCH software to obtain the OTU abundance of each sample. Sequences were deposited in the National Center for Biotechnology Information BioProject repository (accession number PRJNA1007787) and can be accessed through the following link: https://www.ncbi.nlm.nih.gov/bioproject/PRJNA1007787.

### 2.4. Bioinformatics analysis

In this study, we calculated α-diversity, bacterial richness (out numbers), Shannon index, and species evenness (J) based on the OTU table. The Wilcoxon test was used to analyze differences between patients with UC and healthy controls. Additionally, we visualized the differences in microbial community structure (β-diversity) among samples using principal coordinate analysis (PCoA) based on Bray–Curtis distances. Statistical differences were analyzed using permutational multivariate analysis of variance (ANOVA). The Metafor package was used to perform a meta-analysis of α-diversity and bacterial taxa in the 4 studies using both random-effect (RE) and fixed-effect (FE) models. The odds ratio (OR) was calculated based on designated positive values (above the median).

To predict metagenomic function content, the Phylogenetic Investigation of Communities by Reconstruction of Unobserved States (v.1.1.3) was used to predict which genes were present using 16S data. The software utilizes a computational approach to predict the functional pathways from 16S rDNA reads. First, the reads were compared against a reference collection, GreenGenes database, May 2013 version, and the closed-reference OTU table was built using Quantitative Insights Into Microbial Ecology (v1.9.1). The resulting OTU table was normalized by normalize_by_copy_number.py, and Kyoto Encyclopedia of Genes and Genomes (KEGG) and Clusters of Orthologous Groups (COG) metabolic pathways were obtained by metagenomic prediction using predict_metagenomics.py. Statistical difference analyses were performed using ANOVA. The results were visualized using a custom R script based on ggplot2 (v3.0.0).

### 2.5. Random-forest classifiers

Random-forest models were trained separately for each study, and the resulting datasets were merged at the OTU and genus levels to assess the predictive ability of a combination of featured taxa for UC. The models were evaluated using leave-one-out cross-validation, and their predictive power was measured using receiver operating characteristic analysis. To improve the accuracy of microbiome signatures for detecting UC, we utilized a 2-step process involving a modeling workflow and thorough external validation. This approach was designed to prevent overfitting and to generate more realistic model accuracy reports. During the initial phase, we ranked the common OTUs and genera based on their relative abundance. To ensure accurate results, we implemented stepwise feature selection with a 10-fold cross-validation. This allowed us to select predictive microbial features and eliminate irrelevant features, thereby avoiding overestimation of our findings. To evaluate the discriminatory power of OTUs and genera, we calculated the area under the receiver operating characteristic curve (AUC) to evaluate the discriminatory power of OTUs and genera. Statistical and correlation analyses were conducted using the R software (v3.5.3). We utilized the ggplot2 (v3.0.0) and gridExtra packages to create the figures.

### 2.6. Statistical analysis

In this study, the normal distribution of data was determined using the Kolmogorov–Smirnov test. Continuous variables with normal distribution are expressed as mean ± standard deviation, while variables with nonnormal distribution are expressed as median (interquartile range). Categorical variables are presented as percentages. The Student *t* test was applied for normally distributed continuous variables, and the Mann–Whitney *U* test was used for nonnormally distributed continuous variables. Statistical significance was set at *P* < .05.

## 3. Results

### 3.1. Characteristics of included researches

**Figure 1.**
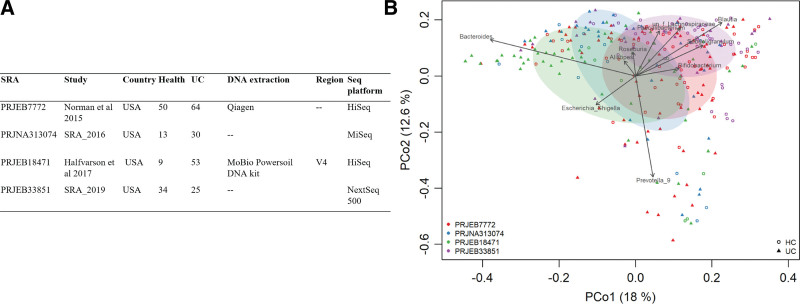
Characteristics of included studies. (A) Characteristics of the studies included in the fecal sample-based analysis. (B) PCoA of all fecal samples at OTU level and the top 10 genera with significant correlations. Each point represents the sample and different shapes display different groups. OTU = operational taxonomic units, PCoA = principal coordinate analysis.

We conducted an analysis based on existing research to characterize the intestinal flora associated with UC. After screening and quality control, we selected 4 studies from the United States, which included 106 healthy individuals and 168 patients with UC. Figure 1 displays the combined samples from these studies, which were analyzed using PCoA at the OTUs level. The composition of gut microbiota in patients with UC was significantly different from that of healthy controls (PERMANOVA, *P* < .01), as determined by PERMANOVA of all samples from the 4 individual studies combined. This difference prompted us to conduct a thorough analysis.

### 3.2. Differences in intestinal microbiota between patients with UC and healthy controls by meta-analysis

**Figure 2.**
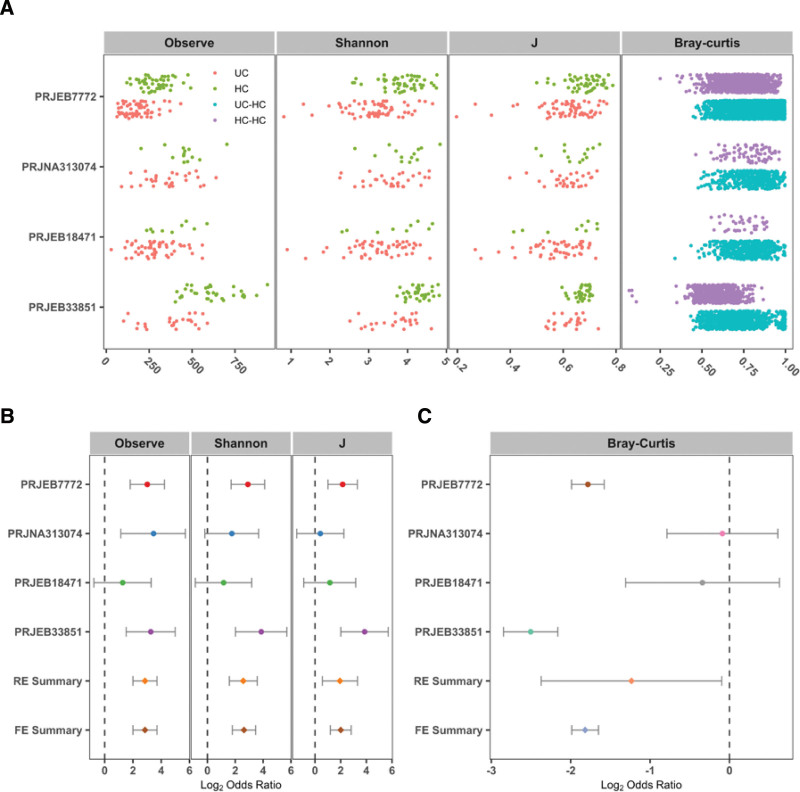
Microbiota diversity between patients with UC and healthy controls. (A) The number of observed OTUs, Shannon index, and evenness in the 2 groups available in 4 studies. (B, C) Forest plot of α-diversity and Bray–Curtis distances between the patients with UC and healthy controls. J = Pielou evenness index, ORs = odds ratios, OTUs = operational taxonomic units, UC = ulcerative colitis.

We conducted a thorough analysis of the intestinal microbiota of patients with UC and healthy controls. Our findings revealed that, with the exception of PRJEB18471, healthy individuals had a significantly higher number of OTUs than UC patients in terms of α diversity. Moreover, within the cohort of 4 studies (PRJEB7772, PRJNA313074, PRJEB18471, and PRJEB33851), our analysis revealed noteworthy variations in the Shannon diversity index, microbial evenness, and Bray–Curtis distances between the healthy control group and patients diagnosed with UC, with the exception of PRJEB18471 and PRJNA313074 (Fig. 2A). In addition, the ORs (calculated by RE model analysis) were higher than 1.0, in both RE and FE models (Fig. 2B). Again, by calculating the ORs based on the Bray–Curtis metric in each study, we found significant bacterial community differences between the healthy control group and patients diagnosed with UC in the FE and RE models (Fig. 2C). The results indicated a significant difference in the indices between the healthy control group and patients diagnosed with UC.

### 3.3. Gut bacterial community structure in patients with UC versus healthy controls and the construction of discriminative models through meta-analysis

**Figure 3.**
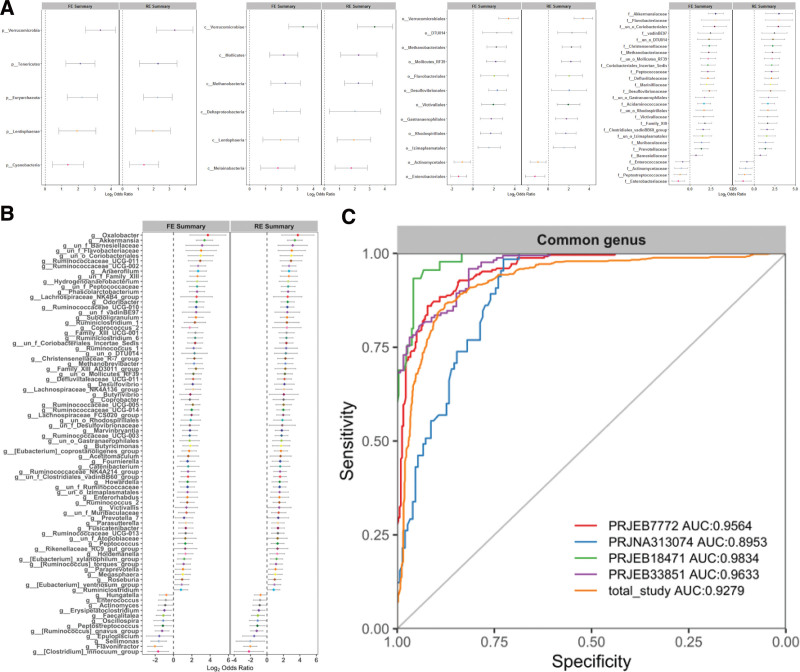
Changes in gut bacterial community composition. (A, B) Relative abundance of microbial taxa at the phylum, class, order, and family genus levels. Forest plot of genera indicated that the 12 genera increased significantly and 70 genera decreased significantly in feces of patients with UC. Error bars depict the 95% CIs. (C) The LOOS performance of genus-level models ranged from 0.8953 to 0.9834. CIs = confidence intervals, LOOS = leave-one-out cross-validation, UC = ulcerative colitis.

To understand the gut flora profiles of healthy individuals and those with UC, we examined the ORs and relative abundance of various bacterial phyla, classes, orders, and families. Our analysis revealed significant differences in 5 phyla, all of which were more abundant in healthy individuals: *Verrucomicrobia, Tenericutes, Euryarchaeota, Lentisphaerae,* and *Cyanobacteria*. At the class level, 6 classes with significant differences were detected and the differential bacteria were significantly enriched in healthy individuals, including *Verrucomicrobiae, Mollicutes, Methanobacteria, Deltaproteobacteria, Lentisphaeria,* and *Melainabacteria.* At the order level, we detected significant differences in the 12 orders between healthy individuals and patients with UC. These orders included *Verrucomicrobiales, DTU014, Methanobacteriales, Mollicutes_RF39, Flavobacteriales, Desulfovibrionales, Victivallales, Gastranaerophilales, Rhodospirillales,* and *Izimaplasmatales*, which were more abundant in healthy individuals. In contrast, *Actinomycetales* and *Enterobacteriales* were significantly enriched in UC patients. At the family level, this study found 27 families with significant differences between healthy subjects and patients with UC. For example, *Akkermansiaceae* was significantly enriched in healthy subjects, whereas *Enterococcaceae* was enriched in UC patients. At the genus level, 82 significantly different genera were detected, with 12 enriched in UC patients and 70 in the healthy control group (Fig. 3A-B). After conducting a pooled analysis, it was found that both analytical models consistently yielded results indicating significant alterations in the structure of gut bacterial communities in patients with UC compared to healthy controls.

To determine whether unique OTUs or genera could differentiate between UC patients and healthy individuals, we created random-forest classifiers. We identified common OTUs and genera that could distinguish between UC patients and controls and then assessed whether the classifier trained in one study could be applied to other studies. We used a cross-validation approach and determined that the AUC values ranged from 0.8953 to 0.9834. After combining the data from 4 studies, the classifier’s efficacy for discriminating patients with UC was 0.9279 (Fig. 3C).

### 3.4. Difference in gut microbiota composition between patients with UC and healthy people in China

**Figure 4.**
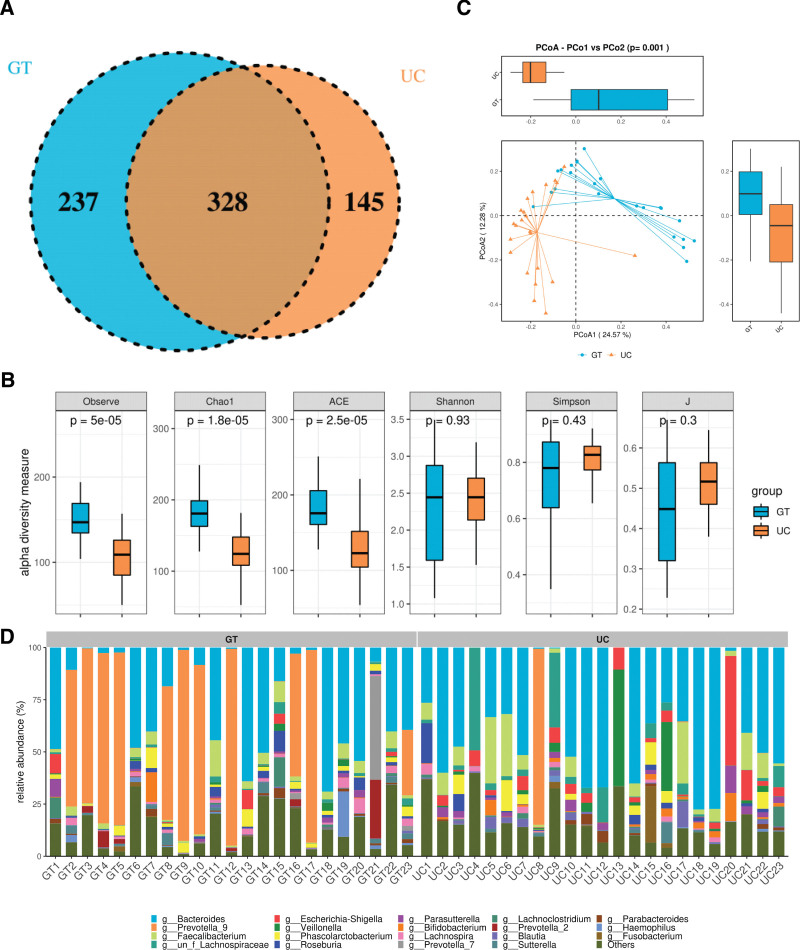
Gut microbiota structure in patients with UC and healthy cohort in China. (A) Venn diagram of shared and unique OTU numbers. (B) PCA scatter plot representing the dispersion of the samples. (C) The α-diversity of bacterial communities. The abscissa represents groupings and the ordinate indicates indices α-diversity indices. (D) Histogram of gut microbiota structure of the top 20 dominant species at the phylum, class, order, family, and genus levels. Different gut bacteria are indicated by different colors; the horizontal axis represents the sample number; the vertical axis indicates the relative abundance of species. OTU = operational taxonomic unit, PCA = principal component analysis, UC = ulcerative colitis.

Although research conducted in the US cohort has shown promising results in discriminating patients with UC, it is imperative to verify whether this classifier is also effective in a Chinese cohort. To achieve this, we collected fecal samples from 23 UC patients and 23 healthy individuals in China and performed 16S rRNA gene sequencing. Our study revealed the presence of 328 bacterial species in both groups. Among these, 237 bacteria were exclusively found in healthy controls, while 145 bacteria were unique to patients with UC (Fig. 4A). Our analysis further revealed that the α-diversity of bacterial communities was significantly higher in the healthy Chinese cohort than in UC patients. Meanwhile, PCoA of OTU-based distances showed that there was a significant difference in the gut microbial distribution between patients with UC and healthy controls in the Chinese cohort (Fig. 4B-C). No significant differences were observed in other α-diversity measures, such as Shannon diversity index, Simpson index, or Simpson evenness, between the 2 groups. However, the relative abundances of the top 20 bacterial communities in the fecal samples showed notable differences at the phylum, class, order, family (Fig. 1, Supplementary Digital Content, http://links.lww.com/MD/L337), and genus levels between the 2 groups. (Fig. 4D).

### 3.5. Taxonomic signatures of gut microbiota in patients with UC and healthy cohort in China

**Figure 5.**
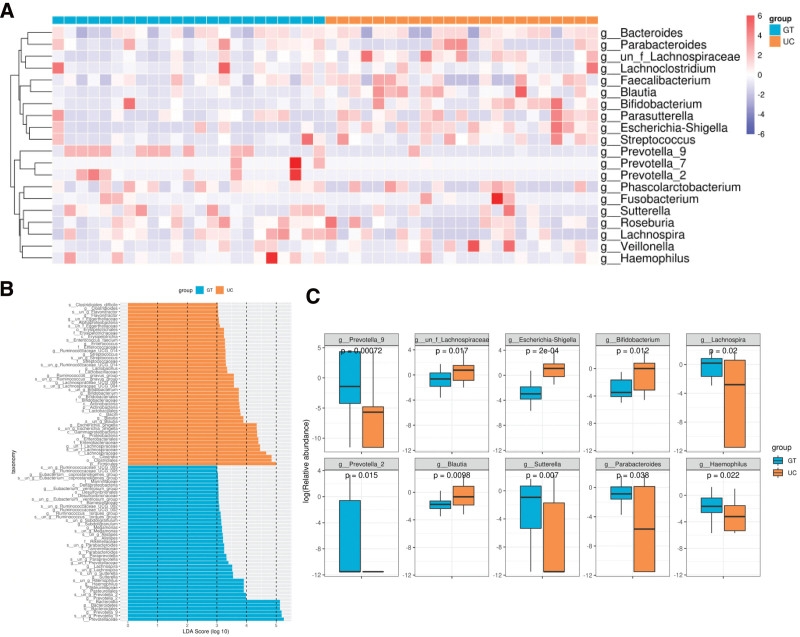
Difference in gut microbiota of patients with UC and healthy people in China. (A) The heat map depicts the relative abundance of the top 20 genera in the 2 groups. (B) The LDA score of the differentially abundant taxa. The cutoff value of LDA was 3.0 or higher. (C) The relative abundances of the top 10 most abundant species showed significant differences between the 2 groups. LDA = linear discriminant analysis.

To compare the gut microbiota of healthy controls and patients with UC in China, we analyzed the distribution of intestinal microflora in both groups. Figure 2, Supplementary Digital Content (http://links.lww.com/MD/L338) shows a heat map of the relative abundance of the gut microbiota at the phylum, class, order, and family levels. At the phylum level, the phyla with the highest abundance were *Lentisphaerae* and *Cyanobacteria*. At the class level, the bacterial phyla with the highest abundance were *Lentisphaeria* and *Melainabacteria*. At the order level, the phyla with the highest abundance were *Bacteroidales* and *Desulfovibrionales*. At the family level, the phyla with the highest abundance were *Prevotellaceae* and *Acidaminococcaceae* (Fig. 2A-B, Supplementary Digital Content, http://links.lww.com/MD/L338). At the genus level, the bacterial phyla with the highest abundance were *Bacteroides* and *Parabacteroides* (Fig. 5A).

**Figure 6.**
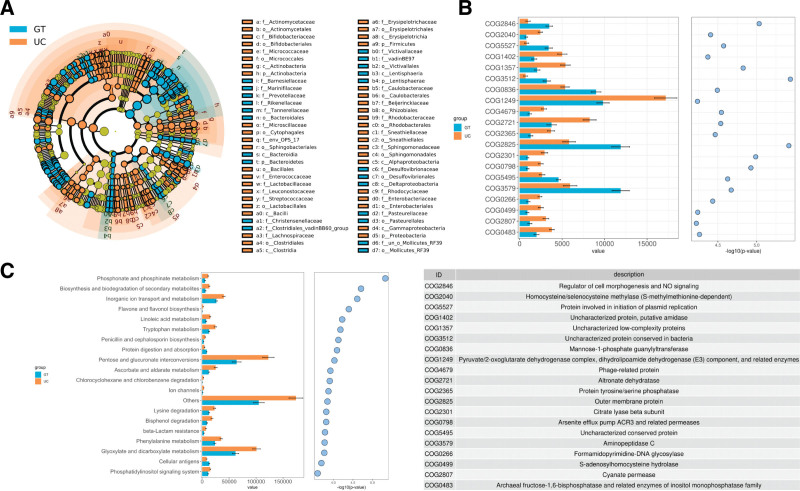
Metabolic pathways of gut microbiota in Chinese UC patients. (A) Taxa significantly associated with UC versus healthy controls, shown in circular cladogram based on the RDP bacterial taxonomy. Each small circle at a different taxonomic level represents a taxon; the size of the small circle diameter is proportional to the relative abundance. Species with no significant differences are indicated in yellow; differential species follow the group for coloration. (B) Pathway analysis based on KEGG annotations. (C) Functional annotation using COG databases. COG = Clusters of Orthologous Groups, KEGG = Kyoto Encyclopedia of Genes and Genomes, RDP = Ribosomal Database Project, UC = ulcerative colitis.

Next, we utilized the linear discriminant analysis effect size method to identify taxonomic biomarkers (linear discriminant analysis > 3.0) present in the gut microbial communities of both patients with UC and healthy controls. The characteristic flora with the highest scores in the UC group were *Firmicutes, Clostridia, Clostridiales, Streptococcaceae*, and *Blautia* (Fig. 5B). The most prominent characteristic flora in the healthy cohort were *P-Bacteroidetes, Bacteroidia, Bacteroidales, Prevotellaceae*, and *Prevotella_9*. Additionally, we obtained the species that were significantly different between UC patients and healthy controls using the Wilcoxon test and showed the top 10 species in terms of mean abundance (Fig. 5C). To gain further insight into the flora found in Chinese patients with UC, the cladogram generated using the linear discriminant analysis effect size method provides a representation of the phylogenetic distribution of taxonomic groups that exhibit differential abundance. Each circle within the cladogram corresponds to a taxonomic level spanning from the phylum to genus (or species) (Fig. 6A).

### 3.6. Fecal metabolomic alterations in Chinese UC patients

Next, to assess the potential correlation between gut microbiota and metabolic pathways in Chinese UC patients, we utilized the COG and KEGG databases to annotate the metabolic and functional pathways of gut microbiome genes (Fig. 6B-C). KEGG pathway enrichment analysis revealed notable differences between the 2 groups. The healthy control group had significantly elevated levels in the protein digestion and absorption pathways, as well as in the cellular antigen pathway. On the other hand, UC patients had significantly upregulated levels in the pentose and glucuronate interconversion pathways, as well as the glyoxylate and dicarboxylate metabolism pathways. Subsequently, COG analysis revealed that the outer membrane protein and aminopeptidase C pathways were upregulated in healthy controls, whereas the pyruvate/2-oxoglutarate dehydrogenase complex and altronate dehydratase pathways were upregulated in patients with UC.

### 3.7. Gut microbiota-based discriminative model for identification of patients with UC

**Figure 7.**
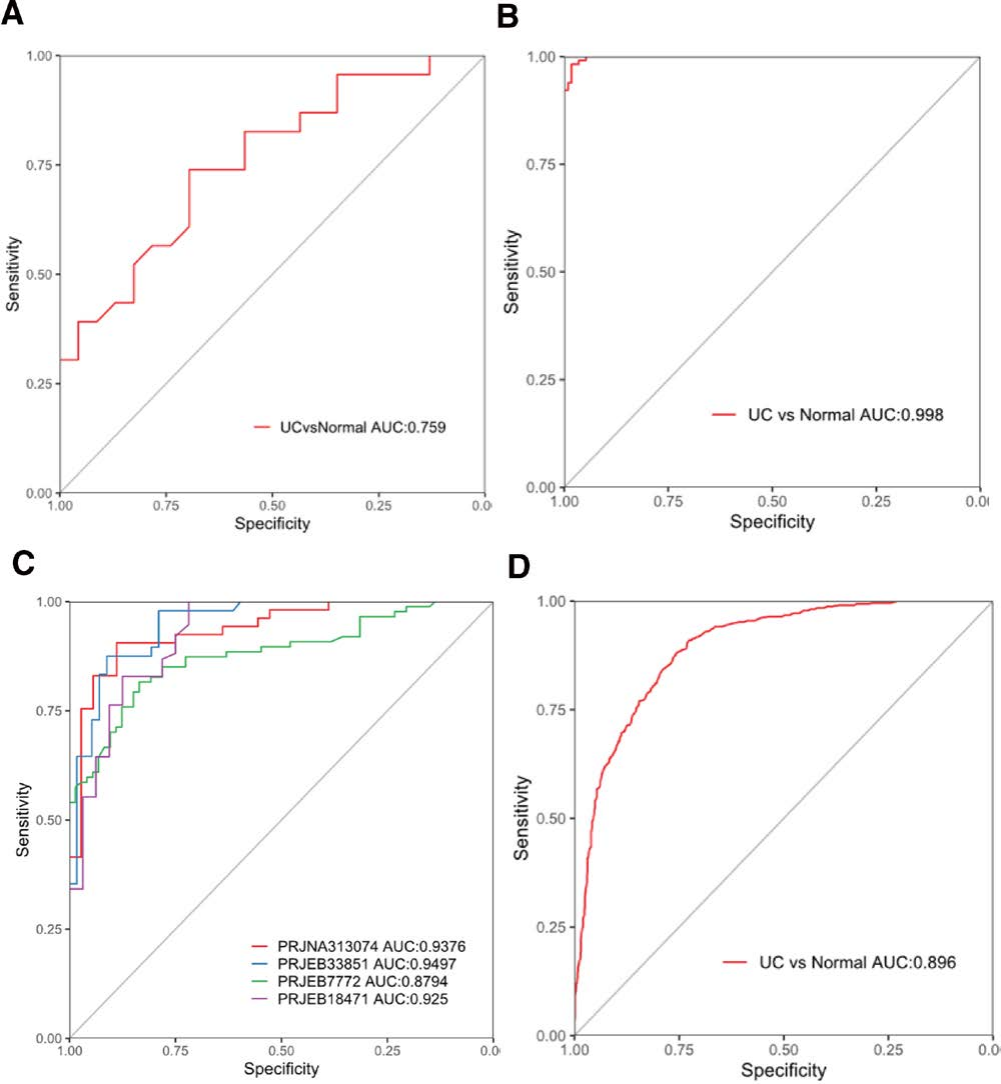
Gut microbiota-based discriminative model for identification of patients with UC. (A) The discrimination efficiency of US-based model to Chinese people. (B) The diagnostic efficiency of China-based model. (C) The model was generated from Chinese UC cohort to analyze American cohort. (D) Comprehensive analysis of Chinese and American cohort to reconstruct the distinguishing model with an AUC of 0.896. AUC = area under the receiver operating characteristic curve, UC = ulcerative colitis.

We developed a gut microbiota-based model with high discrimination efficiency (0.928) in an American cohort. However, when we applied this model to Chinese individuals, discrimination efficiency decreased to 0.759 (Fig. 7A). In the Chinese cohort, a more effective differentiation model was developed, which achieved an impressive AUC score of 0.998 (Fig. 7B). Additionally, the model was applied to an American cohort, resulting in a diagnostic efficiency range of 0.8794 to 0.9497 (Fig. 7C). Next, we conducted a thorough analysis of cohorts from China and the United States. We reconstructed a unique model and identified a new characteristic of intestinal flora as the classification factor. Our classifier achieved an impressive AUC score of 0.896 (Fig. 7D).

## 4. Discussion

The human body contains approximately 1 to 2 kg of microorganisms per adult individual, which has been found to play a crucial role in the host’s overall health and well-being.^[19–21]^ Specifically, studies have extensively explored the regulatory role of gut microbiota in inflammatory bowel disease.^[12,22]^ Observational studies in various countries, including the United States, Europe, Australia, and China, have also shown similar correlations^[1,11,22,23]^; however, differences in diet, culture, and lifestyle habits can limit the generalizability of these findings, as the inherent gut microbiota can vary significantly across different countries and cohorts. The value of intestinal flora is limited for various reasons. To overcome this issue, we analyzed several studies from a US cohort and conducted a meta-analysis. The results showed significant differences in the intestinal flora between patients with UC and healthy individuals, with characteristic genera present in both groups. For example, the higher abundance of *Ackermannia* spp. in the healthy cohort suggests that they may be beneficial bacteria in the host, which is consistent with previous studies.^[24–26]^ The application of these characteristic bacteria can effectively differentiate patients with UC from healthy controls. However, further exploration is needed to establish a causal relationship between the gut microbiota and UC.

To confirm the applicability of the differential diagnostic model in the Chinese cohort, we collected fecal samples from both healthy individuals and patients with UC. We then performed high-throughput detection of intestinal flora and found that the diversity of intestinal flora was significantly lower in patients with UC than in the healthy Chinese cohort. Previous studies have demonstrated that inflammatory bowel diseases, such as UC, can lead to a decrease in intestinal flora diversity, specifically alpha and beta diversity.^[27–29]^ Previous studies have confirmed that UC patients experience a disruption in their intestinal barrier function,^[30]^ which is significantly linked to the loss of gut microbiota diversity.^[31,32]^ A study confirmed significant changes in the abundance of *Firmicutes*, including *Blautia, Clostridium, Coprococcus,* and *Roseburia*, in fecal samples of UC patients in Zhejiang, China.^[33]^ Although the specific types of bacteria identified varied between studies, the overall findings provide strong evidence for the significance of the gut microbiota in the development of UC.

The distribution of intestinal flora varies significantly based on ethnicity and geography.^[34,35]^ To further investigate this, we analyzed data from a Chinese cohort using a classification model based on a US cohort. Our findings showed a decrease in classification efficacy from 0.896 to 0.759, which aligns with our expectation that models based on different cohorts would have lower efficacy when applied to another cohort. To investigate the effectiveness of a widely used classification model, we reconstructed it using data from a Chinese cohort. The resulting model achieves a high classification effectiveness of 0.998. However, when applied to a US cohort, the effectiveness of the model was significantly reduced. After conducting a meta-analysis of the Chinese and US cohorts and developing a classification model, the discriminatory power of the final results was found to be similar to that of individual cohort analysis. However, this did not exceed the highest recorded value. In a previous study on diabetes mellitus, researchers used mixed analysis to identify a range of diabetes-associated bacteria and developed improved classification models,^[36]^ suggesting that utilizing large samples and data from mixed cohorts is a reliable classification strategy.

## 5. Conclusion

Overall, our study revealed notable variations in gut microbiota distribution between individuals with UC and healthy controls. Although the gut microbiota can differentiate between healthy and UC cohorts, the characteristic bacterial taxa is not uniform across the cohorts. Therefore, a thorough analysis of each cohort was necessary before constructing a widely applicable model. We found a significant correlation between the gut microbiota composition and UC, which can serve as a foundation for developing potential strategies for UC prevention and treatment.

## Acknowledgments

We thank all the patients who voluntarily participated in this study.

## Author contributions

**Conceptualization:** Rong Zhang, Jing Chen, Li Liu, Changwei Qiu.

**Data curation:** Rong Zhang, Jing Chen, Li Liu, Xiankun Li, Changwei Qiu.

**Formal analysis:** Rong Zhang, Jing Chen, Li Liu, Xiankun Li, Changwei Qiu.

**Funding acquisition:** Changwei Qiu.

**Investigation:** Rong Zhang, Jing Chen, Li Liu, Xiankun Li, Changwei Qiu.

**Methodology:** Rong Zhang, Jing Chen, Li Liu, Xiankun Li, Changwei Qiu.

**Project administration:** Rong Zhang, Jing Chen, Li Liu, Changwei Qiu.

**Resources:** Rong Zhang, Jing Chen, Li Liu, Xiankun Li, Changwei Qiu.

**Software:** Rong Zhang, Jing Chen, Li Liu, Xiankun Li, Changwei Qiu.

**Supervision:** Changwei Qiu.

**Validation:** Rong Zhang, Jing Chen, Li Liu, Xiankun Li, Changwei Qiu.

**Visualization:** Rong Zhang, Jing Chen, Li Liu, Xiankun Li, Changwei Qiu.

**Writing – original draft:** Rong Zhang, Jing Chen, Li Liu, Changwei Qiu.

**Writing – review & editing:** Rong Zhang, Jing Chen, Li Liu, Xiankun Li, Changwei Qiu.

## Supplementary Material

**Figure:**
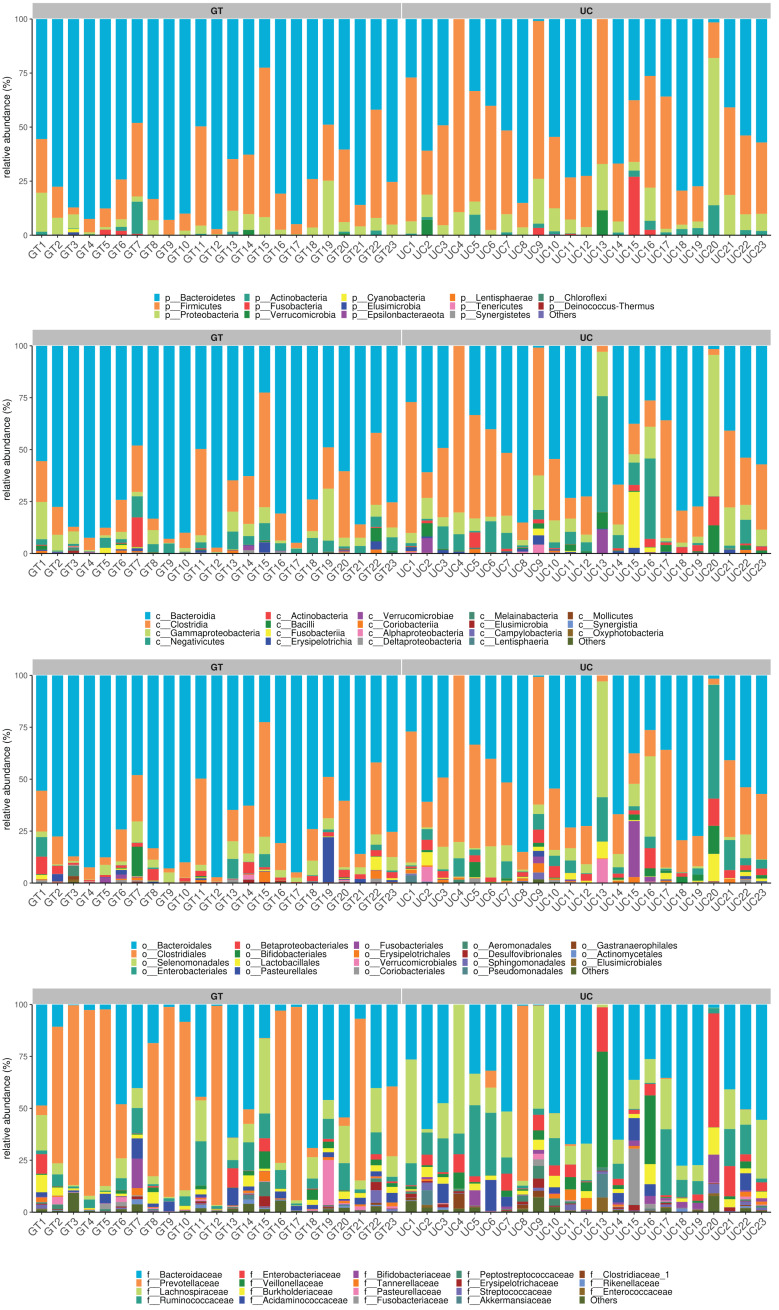


**Figure:**